# A Contrastive Study on Travel Costs of Car-Sharing and Taxis Based on GPS Trajectory Data

**DOI:** 10.3390/ijerph17249446

**Published:** 2020-12-16

**Authors:** Beibei Hu, Yue Sun, Huijun Sun, Xianlei Dong

**Affiliations:** 1 School of Business, Shandong Normal University, Jinan 250358, China; hubeibei@sdnu.edu.cn (B.H.); 2019020901@stu.sdnu.edu.cn (Y.S.); 2 Key Laboratory of Transport Industry of Big Data Application Technologies for Comprehensive Transport, Ministry of Transport, Beijing Jiaotong University, Beijing 100044, China; hjsun1@bjtu.edu.cn

**Keywords:** car-sharing, taxis, travel costs, cost advantages

## Abstract

The emergence and development of car-sharing has not only satisfied people’s diverse travel needs, but also brought new solutions for improving urban traffic conditions and achieving low-carbon and green sustainable development. In recent years, car-sharing has had competition with other ways of getting around, as the acceptance of car-sharing has grown, notably taxis. Therefore, it is particularly important to explore car-sharing travel costs advantages from the perspective of consumers and discover the competitive and complementary spaces between car-sharing and other modes. Therefore, taking Beijing as an example, this paper uses GPS trajectory data based on car-sharing orders to design a travel cost framework of car-sharing and taxis. We calculate and compare the travel cost difference between these two modes under different travel characteristics. The results indicate that car-sharing is a more economical way for consumers to travel for short or medium lengths of time, while people are more inclined to take taxis for distances of long duration. Compared with on workdays, at the weekend, the cost advantage of car-sharing is greater for long-distance trips. Moreover, the cost advantage of car-sharing increases gradually with the increase in travel distance. In addition, the travel costs of car-sharing and taxis are also affected by peak and off-peak traffic periods. Compared with off-peak periods, it is more cost-effective for travelers to take taxis during peak traffic periods for various travel distances. From the perspective of the travel cost, it is of great theoretical significance to discuss the substitution (market competition) and complementary relationship (market cooperation) between car-sharing and taxis in a detailed and systematic way. It provides methods and ideas for the comparative cost calculation of car-sharing and other travel modes. This paper also provides enlightenment and guidance for the development of car-sharing. Enterprises should implement differentiated pricing, designing different charging methods for different traffic periods, travel miles, and rental times, and set up additional stations in the surrounding areas of the city. Relevant government departments should also strictly manage the market access of car-sharing, and add or open car-sharing parking lots in centralized areas and for specific periods.

## 1. Introduction

The sharing economy is said to be among the “10 ideas that will change the world”. In recent years, with the rapid development of the sharing economy, many innovative practices are also making their way into the urban mobile market. They have exerted a profound influence on the economic development of the region, and have also influenced people’s preferences, happiness, and lifestyle to some extent [1]. Car-sharing is an important innovative practice in the urban mobile market; it is a green, convenient, and flexible way to travel. Car-sharing services provide users with car rental services, mainly in the form of self-service vehicle reservation, vehicle pick-up and return, and fees that are charged by the minute or hour. They use mobile Internet, global positioning, and other information technologies to build service platforms. This innovative practice has brought about people’s travel choices [2]. Nowadays, more and more studies have shown that many people are considering giving up their private cars or taxis and turning to car-sharing services [3]. According to statistics, by October 2016, the number of commercial vehicles for car-sharing reached 157,000, providing services to more than 15 million users worldwide [4]. The growing demand for such innovative practice is also being driven by urbanization and the greater flexibility and availability of car-sharing. Car-sharing meets people’s diverse travel needs, helps to ease urban traffic congestion, improves the connectivity of public transport, and promotes the green and low-carbon development of cities. First of all, car-sharing provides people with a new way to make their medium to long distance journeys, especially in urban fringe areas. Compared with bus, subway, and other public transportation modes, car-sharing is more comfortable and flexible. Secondly, car-sharing can meet people’s special travel needs, such as family travel, freight handling, a luxury car experience, etc. However, as a substitute for other ways of urban transportation (especially taxis and online ride-hailing vehicles), car-sharing provides residents with more space for their choice of transportation mode. At the same time, car-sharing also brings greater market competition, which brings some difficulties to urban traffic and market management. Under the current development trend, how to correctly understand the competition and complementary space between car-sharing and other similar travel modes is particularly important.

Car-sharing has developed rapidly in the Chinese market since 2010. In recent years, it has arisen in Beijing, Shanghai, Guangzhou, Shenzhen, Chongqing, and other cities. According to statistics, by June 2018, there were more than 400 registered car-sharing enterprises in China, the number of vehicles in the market exceeded 100,000, and the value of the car-sharing market reached CNY 3.648 billion [5]. With this scale, car-sharing provides multi-level and diverse travel services for the public. It has also improved the travel experience of passengers and become an important choice of the public for traveling under the development concept. As of November 2018, the number of people who had downloaded car-sharing apps reached 9.524 million [6]. The healthy development of car-sharing can effectively control the growth of vehicle ownership, which is an effective way to realize low-carbon and green development in cities. On the one hand, car-sharing can relieve urban traffic congestion and pressure on basic urban transportation facilities (such as parking lots). On the other hand, more than 90% of vehicles in China are new-energy vehicles [7]. This is of great significance for reducing dependence on oil energy, and reducing pollutants and greenhouse gas emissions [8]. With its advantages, the development of car-sharing has been strongly supported by the Chinese government. In 2019, China’s Ministry of Transport and twelve other departments and units formulated the “Green Travel Plan (2019–2022)”. It stressed the importance of encouraging the networked and large-scale development of the car rental industry, further strengthening the promotion and application of energy saving and new energy vehicles in the rental market, and accelerating the construction of charging infrastructure.

Although the emergence of car-sharing has injected fresh blood into the urban traffic market, it has also brought a series of problems. For example, as an alternative to taxis and online car-hailing, the entry of car-sharing has triggered market competition and redistributed the interests of market players [9]. How can we correctly guide the healthy and sustainable development of car-sharing? How can we stabilize the taxi and car-sharing market? These have become important urban traffic governance issues. Correctly understanding the competition and cooperation between car-sharing and taxis (online car-hailing) in the market is a prerequisite for solving the above problems. As far as we know, however, no literature has yet been able to discuss the substitution (market competition) and complementary relationship (market cooperation) between car-sharing and taxis in a detailed and systematic way. Many scholars believe that minimizing consumers’ travel costs (including time and money costs) should be an important goal for the development of car-sharing services in China, along with good service quality (such as comfort and flexibility) [10,11,12]. Therefore, this paper calculates and compares the travel costs of car sharing and taxis under different travel characteristics from the perspective of traveler consumption, focusing on the advantageous market and competitive space of car-sharing. To explore these questions, first of all, we propose a car-sharing and taxi travel costs comparison model (CTTCM). Based on GPS trajectory data, the travel costs of the two modes are calculated. The cost differences for consumers of car-sharing and taxis under different circumstances are compared and analyzed. Then, we explore the advantages of the two modes and focus on the competitive market. Furthermore, under the current prices, the competitive advantages of the car-sharing and taxi markets can be compared, and their substitution (competition) and complementary relationship (cooperation) in the urban transportation market can be understood. Based on the above work, the main contributions of this paper are as follows:

(1) Based on car-sharing’s GPS trajectory data, a travel cost model of car-sharing and taxis is built. The travel costs of consumers using car-sharing and taxis, respectively, are calculated in different travel situations.
(2) Through a comparison of the cost differences of car-sharing and taxis over the time and space dimensions, the conditions under which each has travel cost advantages, and the division of the competitive market, are discussed.
(3) It is found that the two travel modes are both substitutable and complementary under the current price mechanism.

This work has important theoretical significance for the scientific understanding of the competitive patterns and development trends of urban car-sharing and taxis from a cost perspective. It is of great theoretical significance to understand travelers’ preferences under economic rationality and to grasp the influencing mechanism of cost factors on the market demand in the car-sharing market. At the same time, the competitive market can be divided up due to the different advantages of the two modes. This is also of great practical significance for maintaining the stability of the urban taxi (car-hailing) market and promoting the coordination and healthy and sustainable development of car-sharing.

The organizational structure of the paper is as follows: in Section 2, we first review the previous literature on car-sharing. Then, in Section 3, we describe the data and methods. Section 4 focuses on the travel cost advantages of car-sharing and taxis under different travel characteristics. Based on the travel cost advantages of the two modes, the market competition and complementary space are discussed, and policy suggestions are put forward in Section 5. Section 6 draws conclusions, and describes the limitations and future work related to the study.

## 2. Literature Review

As an important mode of urban transportation in the era of the sharing economy and big data, car-sharing has attracted extensive attention since it came into being [13,14]. First of all, the emergence of car-sharing has reduced the number of private cars in cities [15] and has improved traffic conditions and the ecological environment [16,17]. Secondly, in the face of the emergence of car-sharing with positive influences, whether people’s preferences for travel modes will change and the factors influencing people’s choice of car-sharing travel are also very extensive [18,19]. In addition, the widespread use by people and the continuous emergence of car-sharing enterprises have given rise to a series of studies on operation management and vehicle scheduling [20,21]. Therefore, scholars at home and abroad mainly study car-sharing from three aspects: its development advantages, influencing factors that lead people to use car-sharing, and the operation and management of the car-sharing market. They have made a lot of research achievements.

The emergence and development of car-sharing can relieve and improve the traffic and environmental pressure in cities. Car-sharing reduces the number of private cars in cities, thus easing urban traffic congestion. As an important tool, car-sharing has reduced people’s desire to drive, replacing the second and third private cars of the family, thus reducing the mileage of travelers by 15% to 20%, and car ownership by more than 30% [22]. Through surveying car-sharing users in Vancouver, researchers found that whether users use car-sharing as a supplement to other modes of transportation or as a direct alternative to private cars, it can reduce average family car ownership [23]. For example, the two-way and one-way (free-floating) car-sharing services can reduce vehicle ownership by 12% and 35%, respectively. In Beijing, due to the popularity of shared transport modes (including car-sharing and bike-sharing), private car owners decreased by more than 3.2% in 2016 compared with 2015, and people’s travel frequency decreased by more than 55% [24]. The emergence of car-sharing has not only reduced the number of urban cars, but also changed residents’ travel behavior to a certain extent, making people more inclined to adopt greener and more environmentally friendly modes of travel [25]. Car-sharing promotes the development of new-energy vehicles and reduces greenhouse gas emissions, which will reduce carbon dioxide emissions and global warming to some extent [16]. Especially when car-sharing replaces about 10% and about 50% of private cars, the potential of urban traffic to cause global warming drops by about 4% and about 20%, respectively [17]. The development of electric new-energy car-sharing will reduce the use of fossil fuels and greenhouse gas emissions, which is of great significance for improving the urban environment. For example, more than 90% of car-sharing in China uses new-energy vehicles [7]. Chen et al. found that car-sharing had saved about 5% of all energy use and greenhouse gas emissions related to household transportation in the United States [26]. Namazu and Dowlatabadi also proposed that even without reducing the travel mileage, car-sharing could reduce greenhouse gas emissions by more than 30% simply by relying on the characteristics of new energy [27].

Car-sharing travel is affected by many factors, such as travel costs and satisfaction, the city’s scale, user attributes, population density, weather, and air quality [28,29,30,31,32]. Whether residents choose car-sharing depends on their individual attributes. It has been shown that when the search time for car-sharing is short and the charges are low, young middle-income, low-income, and well-educated people are more likely to choose car-sharing to travel, mostly to commute and to go to school and to entertainment venues [33]. Dias et al. [34] found through model estimation that those who were more interested in car-sharing services were mainly young, well-educated, and higher-income people, and their work locations were often in higher density areas. Studies have explained this phenomenon of car-sharing for travel in dense areas by the fact that it can greatly reduce the personal transportation costs of travelers [26]. The use of car-sharing is also affected by uncertainties such as users’ activities, the travel environment, and travel time [35]. In Japan, the wide use of car-sharing has been closely related to car-sharing stations, seasonal impact, age of drivers, and the multimodal transport network (with different transportation modes located close to car-sharing stations) in Japan [36]. In addition, from the viewpoint of government and enterprises, the key factors affecting the development of car-sharing also depend on government subsidies and the operator’s pricing strategy. When the subsidy rate decreases by 2%, the government’s utility fee will increase by 0.13% and the operator’s revenue will decrease by 0.14%, which will interrupt the operator’s plans to improve the service and expand its scale, in turn affecting the sustainable development of car-sharing [37].

For the further development of car-sharing, it is necessary for the government, operators, and other market entities to join forces in its operation and management, and carry out development planning and market management from the aspects of policies, facilities, and funds. First of all, there is still a lot of room for development of the car-sharing market in the future. For example, Chen Xiaohong et al. [38] used the order and user data of EVCARD, the largest car-sharing company in Shanghai, as an example to analyze the characteristics of high-frequency users and commuting users. They found that further development of car-sharing in the future would not put a significant traffic burden on the city. According to a declarative preference experiment conducted by Catalano et al. [39], it can also be inferred that the car-sharing market may grow by 10% in the future, against a background of the implementation of an active policy of restricting private transport. Secondly, in the face of the expanding market, cooperative measures between the government, operators, and other industries will have an important impact on the car-sharing market. A study found that the economic performance of urban car-sharing systems depends on fleet size, station or free-parking-area location decisions, pricing policies, etc., and this finding may be used by the government to strictly estimate the impact on car-sharing usage, and also enable operators to better manage their operating systems [40]. At the same time, in terms of market management, Terrien et al. [21] emphasized the importance of cooperation between the public and private sectors. They argued that both the government and car-sharing companies should have specific management departments which are independent of the car-sharing operations and carry out management innovation. From the perspective of the relationship between the government, business, and travelers, Chou Yang et al. [41] established an enterprise behavior and government subsidies dynamic game model, and analyzed the influence of government subsidies on the development of car-sharing enterprises. The study found that only when there are government subsidies in the car-sharing market is the behavior of enterprises in entering the market rational. Lu Ke et al. [42] established an evolutionary model of the trilateral game between government, enterprise, and traveler, and analyzed the interaction between the decision-making behaviors of the three parties. The study found that in the development of the car-sharing industry, we should attach importance to the relationship between these three. Meanwhile, we can take measures to promote the balanced and healthy development of the car-sharing market. In addition, some scholars have carried out studies on delicate management and operational aspects, such as the configuration of charging stations, the location of stations, and the optimization of fleet numbers. For free-floating car-sharing, which has emerged in recent years, the downtime caused by charging has become a major barrier to sustainable operations. In order to solve this management problem, Mohammad S. Roni et al. [43] studied the relationship between the stop time of fleet vehicles and the number of charging stations. They optimized the distribution, number, and location of charging stations at the same time. The average downtime of a car-sharing fleet caused by charging and waiting is also closely related to the number of charging times. Bauer et al. [44] modeled the fleet operation of car-sharing service providers to rationally configure stations, taking these characteristics into account. Yang et al. [45] used a data-driven optimization method to determine the location of car-sharing charging stations in a city and minimize the cost of government and operator investment in infrastructure. All of this research has played a positive role in improving the operating efficiency of car-sharing.

To sum up, previous studies have mostly focused on the development advantages of car-sharing, the influencing factors in car-sharing travel, and the operation and management of the car-sharing market. However, few scholars have explored the competition and cooperation relationship between car-sharing and other modes of travel from the perspective of travel cost. On the one hand, car-sharing and taxis share a high degree of similarity in terms of being motorized travel, and there is a large degree of market substitution between them. On the other hand, under the current pricing mechanism, the two modes have their own competitive advantages and complementary markets. From the perspective of travel costs, this paper constructs a comparison model of the travel costs of car-sharing and taxis. Then, we take Beijing as an example to calculate and compare the cost differences and cost advantages for consumers who use car-sharing and taxis in different situations. The purpose of this study is to explore the advantages of car-sharing and taxis from the perspective of travel costs and a focus on the market. This study provides theoretical support for urban traffic management and planning, which will be of positive significance to the healthy and sustainable development of the urban car-sharing and taxi markets.

## 3. Methodology

**Figure 1:**
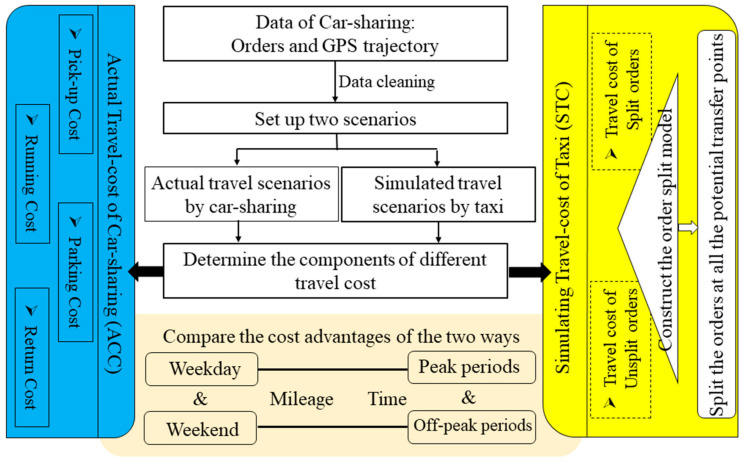
Framework of research methodology.

In this section, we propose a new model called CTTCM (car-sharing and taxi travel costs comparison model). In this model, we construct the travel costs framework from the perspective of a traveler and determine the cost composition of car-sharing and taxi travel under the same travel situation, respectively. Many of the previous travel costs estimates were from the perspective of operators and they did not take into account the opportunity costs of travelers [46]. Our model quantified opportunity cost such as time-cost as the money-cost. Then, when we calculate the taxi costs, it is different from the previous direct calculation [47,48]. Our model simulates the taxi travel scene more realistically and conforms to the basic characteristics of the taxi mode. Therefore, we split the order under the simulated taxi travel scenario, and then, add up the taxi travel costs. Figure 1 shows the research process.

### 3.1. Data Source and Preprocessing

The research travel data used in this paper are provided by Beijing Automotive Group, the operator of the MoreFun car-sharing platform, and range from 1 July 2017 to 30 September 2017. The travel data include 51,250 travel orders of 27,977 cars and their trajectory data. The order data for car-sharing (as shown in Table 1) are composed of Order ID (desensitized), Order time stamp, Pick-up time stamp, Return time stamp, Pick-up station, Return station, CAR_ID, and other fields. Each order corresponds to multiple trajectories. The trajectory data (as shown in Table 2) consist of six fields: CAR_ID, DEVICE_NO, Time stamp (of trajectory point), Longitude (coordinates), Latitude (coordinates), and Instantaneous speed.

The car-sharing orders and trajectory data are cleaned, including the deletion of abnormal and unreasonable data. Abnormal orders are mainly caused by system and data collection errors, such as return times earlier than pick-up times, pick-up or return stations not registered among the company’s stations, vehicles not registered in the vehicle registration table, etc. Unreasonable data refer to car-sharing orders whose time of use exceeds 96 h (the company stipulates that cars can be rented for no more than 96 h at a time), travel distance is less than 1km, or trajectories are beyond the latitude and longitude coordinates of the Beijing area’s trajectory data. Following this, we have 42,807 valid car-sharing orders remaining.

Using the GPS trajectory data for the car-sharing, we then calculate the total travel time, travel distance, travel time at low speed (when the instantaneous velocity is less than 12 km/h), night driving time (23:00 (incl. 23:00 p.m.) to 5:00 a.m. the next day (excl. 5:00 a.m.)), and the peak and off-peak traffic driving times (peak hours are 7:00 a.m.–9:00 a.m. in the morning and 17:00 p.m.–19:00 p.m. in the afternoon/evening). At the same time, we record the location and time of each order.

### 3.2. Methods

**Figure 2:**
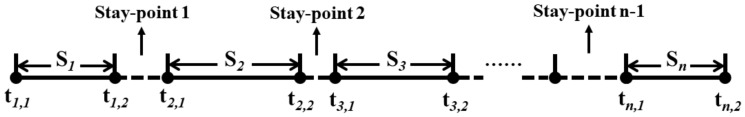
An example of the order trajectory data of car-sharing.

Consider the trajectory data for a car-sharing order *A* = {*S*_1_, *S*_2_, …, *S*_n_}, shown in Figure 2. Typically, a car-sharing order consists of several “stay-points”, by which we mean periods during which the car is stationary. Let *S_k_* (*k* = 1, 2, …, *n*) represent the kth “continuous driving period” of a car-sharing order, *t_k_*_,1_ and *t_k_*_,2_ represent the start-time and end-time of this period. Thus, *t_k,_*_1_ − *t_k_*_−1,2_ can represent the time length of the k−1th stay-point of the order, which is denoted by $\Delta t_{k-1}$.

Firstly, we use the charges for car-sharing in Beijing to calculate the actual travel cost of car-sharing (ATC) based on the trajectory data from the car-sharing orders. Next, we propose a taxi travel cost estimation model to simulate and estimate the travel costs of taxis (STT) based on the trajectory data from the car-sharing orders. Finally, we compare and analyze the difference in travel costs between car-sharing and taxis.

#### 3.2.1. Calculating the Actual Travel Cost of Car-Sharing (ATC)

The ATC ($C_{share}$) is composed of four parts: the pick-up cost ($C_{s\_get}$), the driving cost ($F_{share}$), the parking cost ($C_{s\_park}$), and the return cost ($C_{s\_return}$), as shown below:

(1) $$C_{share}=C_{s\_get}+F_{share}+C_{s\_park}+C_{s\_return}$$

The pick-up cost refers to the cost of traveling from the origin point to the nearest pick-up station to pick the car up. It includes the cycling cost (such as a bicycle or electric bike) and the walking cost, and is calculated as follows:

(2) $$C_{s\_get}=w_{i}C_{s\_get}^{i}+w_{j}C_{s\_get}^{j}$$

where i,j represent cycling and walking, $w_{i}$ and $w_{j}$ represent the share of cycling and walking done when traveling to pick the car up, $C_{s\_get}^{i}$ represents the cycling cost, using bike sharing, and $C_{s\_get}^{j}$ represents the walking cost (which is an opportunity cost). The calculation formula for the last two costs is as shown in Formula (3):

(3) $$\left\{\begin{array}{l} C_{s\_get}^{i}=c\frac{l_{i\_get}}{v_{i}}+F_{get\_ride},\;C_{s\_get}^{j}=c\frac{l_{j\_get}}{v_{j}}\\ l_{i\_get}=\frac{1}{4}\min\{L_{i}^{\alpha \beta }\},\;l_{j\_get}=\frac{1}{4}\min\{L_{j}^{\alpha \beta }\}(\alpha ,\beta =1,2,3,\cdots ,211\;\&\;\alpha \neq \beta ) \end{array}\right.$$

where c is the travelers’ income per unit time, and $l_{i\_get}$ and $l_{j\_get}$ are the traveler’s cycling and walking distances. Since the trajectory data only record the travelers’ trajectories from the pick-up station to the return station, we cannot observe the path from the origin to the pick-up station. Therefore, in this paper, we use a quarter of the distance from the pick-up station to the next nearest pick-up station as the traveler’s average pick-up distance. α and β represent 211 pick-up and return stations. $L_{i}^{\alpha \beta }\;and\;L_{j}^{\alpha \beta }$ represent the actual distance for the traveler from station α to station β (obtained using the Gaode open platform for walking and cycling path planning). $v_{i}$ and $v_{j}$ refer to the average speeds of riding and walking. $F_{get\_ride}$ refers to the charge made by the bike-sharing platform for users who ride to the pick-up station.

Next, the driving cost ($F_{share}$) refers to the charge to the user for using the car-sharing service (not considering car damage, traffic accidents, etc.). It includes a time charge and a mileage charge, which are calculated as shown in Formula (4):

(4) $$F_{share}=p_{t}T_{share}+p_{s}S_{share}$$

where $p_{t}$ represents the driving cost per unit of time, $p_{s}$ represents the driving cost per unit of mileage, $T_{share}$ represents the total rental time in the car-sharing order, and $S_{share}$ represents the total mileage traveled in a car-sharing order.

It can be seen from the car-sharing trajectory data that there are multiple stay-points in a car-sharing trip, with different stay durations. This shows the obvious “segmental travel” characteristic. According to the statistics, there are 93,911 stay-points that last longer than 15 min in the trajectory data, with an average of 2.2 stops per order. According to the standard parking charge on Beijing municipal roads, parking fees will be charged for any incident of parking that lasts more than 15 min [49]. Thus, the parking-cost ($C_{s\_park}$) refers to the charges for parking incurred during the use of a car-sharing service. The calculation of this is as shown in Formula (5):

(5) $$C_{s\_park}=\sum_{\lambda =1}^{3}(p_{\lambda }\sum_{k=1}^{n}t_{k}^{\lambda })$$

where $\lambda \left(\lambda =1,2,3\right)$ represents parking fee in zones I, II, and III in Beijing. Here, zone I includes four key areas: the area within the Third Ring Road (incl.) and the Central Business District (CBD), Yansha area, the West district of Zhongguancun Science Park, and Cuiwei Business District; zone II includes the area within the Fifth Ring Road (incl.) excluding the areas in zone I; zone III refers to the other area outward the Fifth Ring Road in Beijing. $p_{\lambda }$ is the price of parking in zone λ. $t_{k}^{\lambda }$ represents the kth time the user parks in zone λ ($t_{k}^{\lambda }\geq 15$ mins, k=1,2,⋯,n).

The return cost ($C_{s\_return}$) refers to the cost to the user of traveling to their destination from the nearest return station. According to the statistics from the car-sharing orders used in this study, in 91.2% of the orders, the pick-up station is the same as the return station. In this paper, we therefore set the pick-up cost equal to the return cost, that is $C_{s\_return}=C_{s\_get}$.

#### 3.2.2. Simulating the Travel Cost of a Taxi (STT)

To simulate the travel cost of a taxi, the following assumptions are made in this paper:

(1) We assume that travelers behave like “homo economicus”, in that they will choose the cheapest transfer method to complete their journey.
(2) We do not distinguish between the driving experience of taxi drivers and car-sharing drivers as regular car-sharing drivers can use an onboard navigation system, which can help them choose the optimum travel route (shortest or congestion-avoiding) if they are not familiar with the route. Thus, we assume that both car-sharing drivers and taxi drivers will choose the most efficient route when driving. In other words, they will choose the same routes for the same travel in our research.
(3) The trajectory data for the car-sharing contain multiple stay-points, which we assume to be 15 min or longer in duration (since 15 min is the time unit used in Beijing parking fees).
(4) We assume that each order is a solo trip. In other words, the transfer cost only considers the cost of one person, not the case of group travel.
(5) We do not consider the social costs of travelers when calculating, including the potential costs of traffic congestion, environmental pollution, travel safety, etc.

Next, we build the taxi travel cost calculation model [50], and use the car-sharing trajectory data to calculate the STT in the taxi travel scenario.

##### Step 1: Split Up the Car-Sharing Orders

There is a significant difference between travel by car-sharing versus taxis. Generally speaking, a car-sharing order will often have multiple long-term stay-points (of more than 15 min). Due to the impact of having to travel to and from pick-up and return locations, car-sharing travelers will not make frequent returns and pick-ups. On the contrary, when travelers take taxis, there are usually no long stay-points because travelers can get in and out of taxis whenever and wherever they want, and extra low-speed driving costs will be incurred if taxis have to wait for them at stay-points. In particular, when the taxi mileage increases, there is an additional remote charge.

In order to replicate a car-sharing order using the taxi travel mode, it is necessary to make the order travel in line with the characteristics of the taxi travel scenario (without long stay-points). First of all, we regard all stay-points in the car-sharing orders as possible taxi transfer points for travelers. When a traveler is going to stop for too long, they will be inclined to change taxis, that is, to re-order another taxi. In other words, when we use taxis to replicate a car-sharing order, we need to split the original order into several taxi order sets. We split the car-sharing order according to the principle of minimum cost.

**Figure 3:**
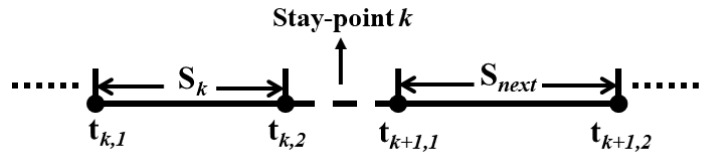
Two trajectories before and after stay-point *k* in a car-sharing order.

When the transfer cost of traveler $F_{1}$ at a stay-point is more than the no-transfer cost $F_{2}$ (Transfer cost refers to the travel cost from this stay-point to the next stay-point. It includes the waiting time cost at this stay-point plus the driving cost of the next continuous driving period), the traveler chooses the non-transfer mode for the next continuous driving period $S_{next}$. Instead, the traveler chooses to take another taxi to complete the next continuous driving period $S_{next}$. The car-sharing order will therefore be split into two taxi sub-orders. Take two consecutive travel trajectories before and after stay-point k as an example (as shown in Figure 3). The calculation methods of the passenger’s transfer and non-transfer costs at the stay-point are shown in Formulas (6) and (7):

When $0<S_{next}\leq s$,

(6) $$\left\{\begin{array}{l} F_{1}=cT+F_{a}\\ F_{2}=F_{low\_speed}+p_{b}S_{next} \end{array}\right.$$

When $S_{next}>s$,

(7) $$\left\{\begin{array}{l} F_{1}=cT+F_{a}+p_{b}(S_{next}-s)\\ F_{2}=F_{low\text{-}speed}+p_{b}S_{next} \end{array}\right.$$

where $F_{a}$ is the base rate, $P_{b}$ is the unit price of mileage, $F_{low\text{-}speed}$ is the waiting cost of the taxi when the traveler does not transfer at the stay-point (charge based on the standard low-speed taxi fee), s is the service mileage within the base rate, T represents the time it takes for a traveler to transfer to another taxi, c is a traveler’s income per unit of time, and cT represents the monetary cost of transferring to another taxi.

##### Step 2: Calculate the Total Simulated Travel Costs of Taxis after the Order Split

**Figure 4:**
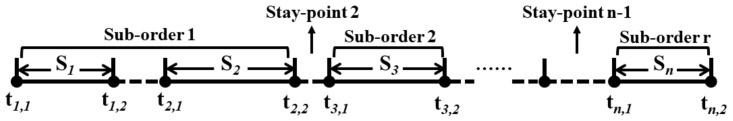
A car-sharing order split into r sub-orders.

After a car-sharing order has been split as in Step 1, it consists of multiple taxi orders, as shown in Figure 4. Next, we calculate the taxi travel costs of all sub-orders and add them up according to the standard charges for taxis.

For example, a car-sharing order m has been split into r sub-orders, expressed as $D_{u}(u=1,2,\cdots ,r)$. According to the standard charges for taxis, we calculate the taxi travel cost $F_{D_{u}}(u=1,2,\cdots ,r)$ of every sub-order. Finally, the taxi travel cost of the order can be calculated by Formula (8):

(8) $$F_{m\_taxi}=(r-1)cT+\sum_{u=1}^{r}F_{D_{u}}$$

where cT is the money of the traveler transfer to another taxi.

##### Step 3: Identify Transfer Points and Calculate Total Cost for Unsplit Car-Sharing Orders

**Figure 5:**
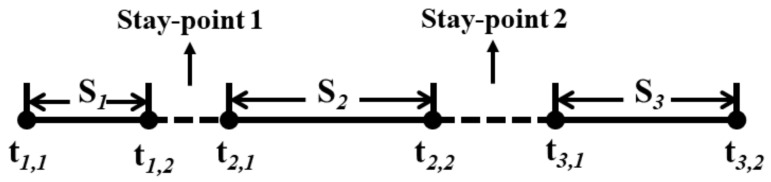
A car-sharing order including two stay-points (possible transfer points).

When a car-sharing order is not split, the stay-stops in those orders may also become potential transfer points. Next, we take a car-sharing order with two stay-points (possible transfer points) as an example (as shown in Figure 5). The following three situations should be considered when calculating the STT:

(1) The traveler does not transfer at the stay-points. When the traveler takes the same taxi to complete the journey, $S_{1}+S_{2}+S_{3}$, the total travel cost of the taxi is recorded as $G_{1}$.
(2) The traveler transfers at one of the stay-points. If the traveler transfers at stay-point 1 but does not transfer at stay-point 2, the traveler takes two taxis to complete the journey, which now consists of $S_{1}$ and $S_{2}+S_{3}$. The travel cost of the taxis is recorded as $G_{2}$. If the traveler transfers at stay-point 2 but not at stay-point 1, the traveler also takes two taxis to complete the journey, which is now $S_{1}+S_{2}$ and $S_{3}$. The travel cost of the taxis is recorded as $G_{3}$.
(3) The traveler transfers at both stay-points 1 and 2. The traveler takes three taxis to complete the journey, $S_{1},S_{2},S_{3}$. The total travel cost of the taxis here is recorded as $G_{4}$.

According to the principle of minimum cost, the potential travel cost of the taxis, named $F_{taxi}$, is shown in Formula (9) based on a car-sharing order with two stay-points:

(9) $$F_{taxi}=\min\{G_{1},G_{2},G_{3},G_{4}\}$$

Therefore, the STT, which is the simulated cost of a user taking taxis (or one taxi) to travel, is calculated by Formula (10), which consists of the split order cost and the unsplit order cost:

(10) $$C_{virtual\text{-}taxi}=\left\{F_{m\_taxi},F_{taxi}\right\}$$

## 4. Results

In order to compare the ATC and STT, we now quantify the cost advantages of the two travel modes. We make subjective prior assumptions about some model parameters when calculating the ATC and STT:

(1) The average monthly salary of employees in Beijing in 2017 was CNY 8467, as reported by the Beijing Municipal Human Resources and Social Security Bureau. It is calculated that the per capita income per minute in Beijing is about 0.8 CNY/min. We thus assume c=0.8 CNY/min in our paper.
(2) When the travelers pick up or return a car by walking or riding, we assume a riding speed of $v_{i}=15$ km/h and a walking speed of $v_{j}=4.5$ km/h.
(3) We set the ratio of riding to walking as 1:1 when travelers are picking up and returning cars—that is, $w_{i}=w_{j}=\frac{1}{2}$
(4) The charge for bicycles on the bike-sharing platform is CNY 1 per 30 min.
(5) According to the standard prices of Beijing Automotive Group for car-sharing, the price per unit of time is $p_{t}=0.17\;\mathrm{yuan}/\min\mathrm{ute}$ and the price per unit of mileage is $p_{s}=1$ CNY/km.
(6) According to a survey, the minimum waiting time for taxi passengers in Beijing is 5 min. Therefore, it is assumed that the time cost to passengers of transferring to another taxi is T=5 mins.
(7) The standard charges for parking in Beijing are shown in Table 3.
(8) The standard charges for taxis in Beijing are shown in Table 4.

### 4.1. Overview

**Figure 6:**
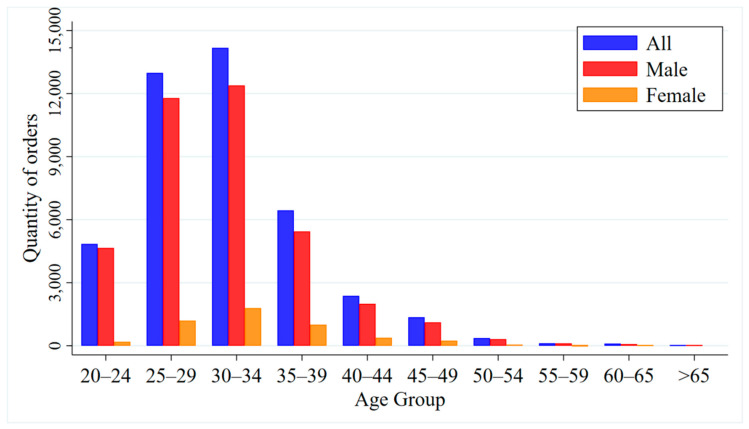
Age and gender distribution of people who booked car-sharing orders.

Figure 6 shows the age and gender distribution of car-sharing users. From the perspective of gender, males are relatively more likely to use car-sharing, accounting for 88.6% of the total. This is mainly because there are far more men with driving licenses than women in China (according to statistics, the ratio of men to women with driving licenses was about 7:3 in 2018 [51]). Additionally, in terms of driving skills and experience, male drivers tend to have better adaptability than female drivers. From the perspective of age, more young people like to use car-sharing. For example, users who are 25–34 years old account for 63.5%. This is because with the rapid development of mobile Internet, the convenience and flexibility of car-sharing makes it more acceptable for young people. This also reveals that car-sharing in China is applicable to a small number of people, and the mode of travel and usage is less attractive to middle-aged and elderly people [52]. In addition, it can better meet the daily commuting and travel needs of this age group [53]. On the contrary, there are relatively few middle-aged and elderly people who use car-sharing. The proportion of users over the age of 50 is only 11.3%.

**Figure 7:**
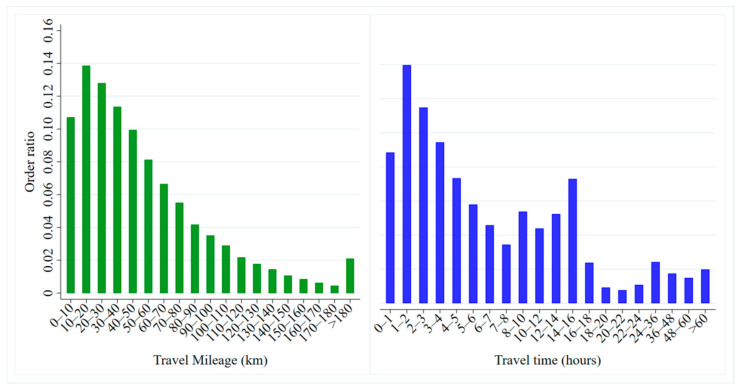
Mileage and travel time distributions of car-sharing orders.

Figure 7 shows the distribution of the mileage and travel times of the car-sharing orders. On the whole, car-sharing orders are mainly concentrated around 20 km in distance, and 2 h in time. When the travel distance is further than 10 km, the quantity of car-sharing orders gradually decreases with the travel mileage. Orders with travel distances of 10–50 km account for about 48% of all orders. Those of 10–20 km are the most common, accounting for 13.9%. From the perspective of travel time, there are two obvious peaks, 1–2 h and 14–16 h, accounting for 14% and 7.3%, respectively. When the travel time is between 16 and 22 h, the order quantity gradually decreases as the time increases. The reason is that the charges for mileage and hours result in high hourly charges when the time period is nearly a day’s rental. Therefore, fewer users choose to travel by car-sharing in such cases.

**Figure 8:**
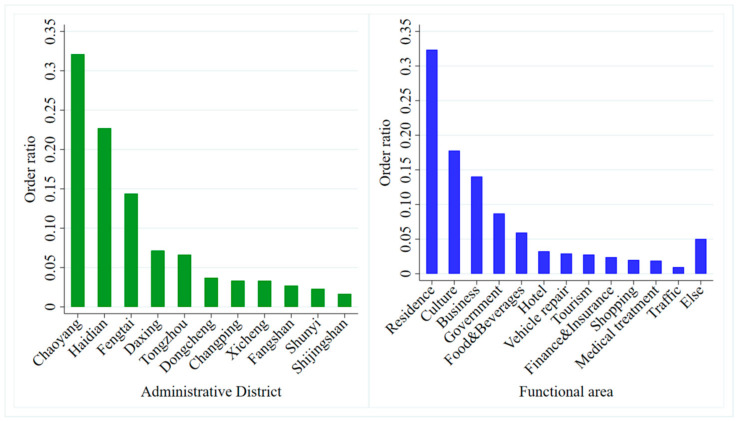
Administrative district and functional region distributions of car-sharing orders.

Figure 8 shows the distribution of car-sharing orders by administrative district and functional area (point of interest, POI). In terms of administrative districts, Chaoyang, Haidian, and Fengtai have the most orders, accounting for about 70% of all orders. Chaoyang district has the largest number of orders, accounting for 32.1%. This is mainly caused by the fact that the Chaoyang district has the largest number of pick-up and return stations, with 30.8% of all the stations in Beijing. In terms of functional areas (POIs), commercial residence areas, science, education and culture areas, and company areas have the largest numbers of car-sharing orders, accounting for 64.2% of the total. Among these, the commercial residence functional areas have many more orders than the others, accounting for 32.4%. Young people tend to work in commercial residences. They need convenient and flexible transportation for daily commuting and official travel. Moreover, they are more likely to accept the concept of shared travel modes. According to the above results, the use of car-sharing is limited by the geographical location of stations. This problem has also been a limitation of the expansion of the market for station-based car-sharing.

### 4.2. Calculating and Comparing the ATC and STT

**Figure 9:**
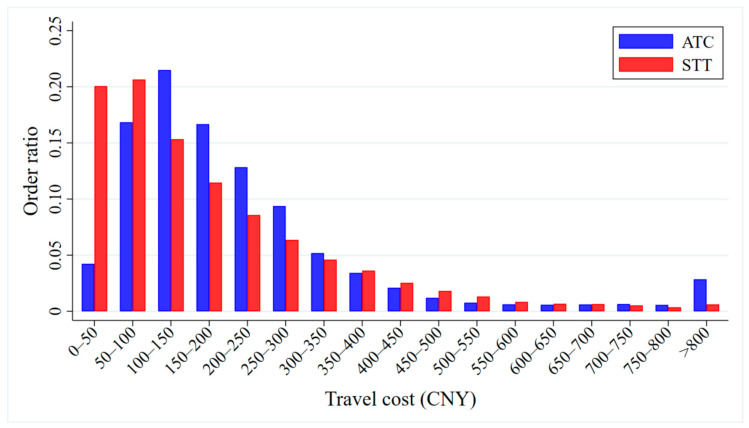
Distribution of the actual travel cost of car-sharing (ATC) and the travel costs of taxis (STT).

We calculate the ATCs and STTs of the car-sharing orders and present their cost distribution characteristics in Figure 9. In general, 84.9% of the ATCs are between CNY 50 and 400. Among them, the largest numbers are distributed in the range of CNY 100–200, accounting for 35.2% of the total orders. About 95% of the STTs are within the range of CNY 0–500. STTs of less than CNY 100 make up the largest share, accounting for 40.7% of all orders.

Looking at a travel cost of less than CNY 100, there are relatively more orders with STTs in this part of the distribution than orders with ATCs in this part. Especially when looking at a travel cost of between CNY 0 and 50, there are about six times as many STTs as ATCs. However, between CNY 100 and 400, all of the buckets have more orders with ATCs in that range than STTs. For travel costs of CNY 400 to 800, the numbers of orders are roughly the same for both ATC and STT. For travel costs of more than 800 yuan, there are five times as many orders with ATCs in that region as STTs.

### 4.3. Comparison of ATC and STT on Workdays and Weekends

**Figure 10:**
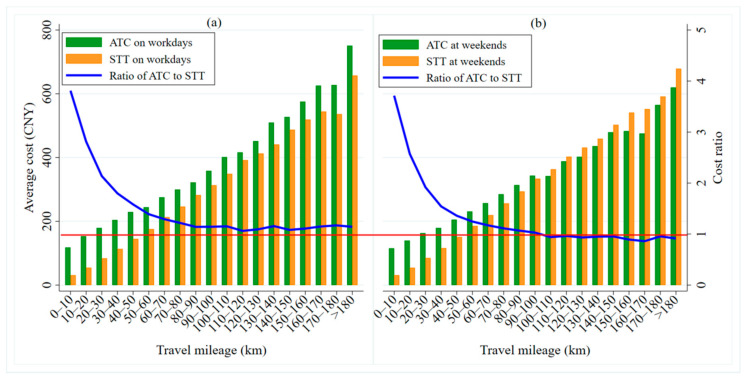
ATC and STT distributions over travel mileage on workdays and at weekends. (**a**) shows the ATC, STT and cost ratio of them over mileage on workdays, while the (**b**) is at weekends.

The travel costs of car-sharing and taxis are affected by whether it is a workday or a weekend. Figure 10 shows the distributions of ATC and STT over different travel distances on workdays and weekends. Overall, the greater the travel distance, the higher ATC and STT are. With an increase in travel distance, the cost ratio (ATC/STT) between taxis and car-sharing decreases gradually (as shown by the blue line in Figure 10a,b).

As shown in Figure 10a, the ATC is higher than the STT for different travel mileages, which indicates that consumers have more cost advantages when traveling by taxi on workdays. When the travel distance is more than 100 km, the cost ratio gradually approaches 1 and then, tends to be stable (as shown by the red horizontal line in Figure 10a). When traveling short distances (less than 10 km), the cost advantage of taking a taxi is obvious, as the ATC is 3.8 times higher than the STT. For long journeys (of more than 100 km), the cost ratio between these two modes is relatively lower, with the ATC being only about 1.1 times the STT. On the weekend (as shown in Figure 10b), when the travel distance is less than 100 km, it is more cost-advantageous for travelers to travel by taxi (the ATC is higher than the STT). On the contrary, when the travel distance exceeds 100 km, it is more cost-advantageous for travelers to use car-sharing, shown by a cost ratio below 1, specifically about 0.9 (as shown by the red horizontal line in Figure 10b).

On workdays and at weekends, we have found that taxis are a more economical way to travel on short and medium-length journeys. This is mainly because most people traveling these distances are making single-target journeys, such as for commuting, leisure and entertainment, shopping, etc. Single-target travel does not require wait or transfer costs, making the total travel costs of the taxis less. If travelers use car-sharing to travel, they need to register, make an appointment, check in, pick-up, and return the car. These complex processes take up travelers’ time and impose some costs. When traveling long distances on weekends, people are more likely to choose car-sharing. This is mainly because travelers often take self-drive trips and do other activities to relax. For young carless families, comfortable, flexible, and green car-sharing becomes their first choice [54].

**Figure 11:**
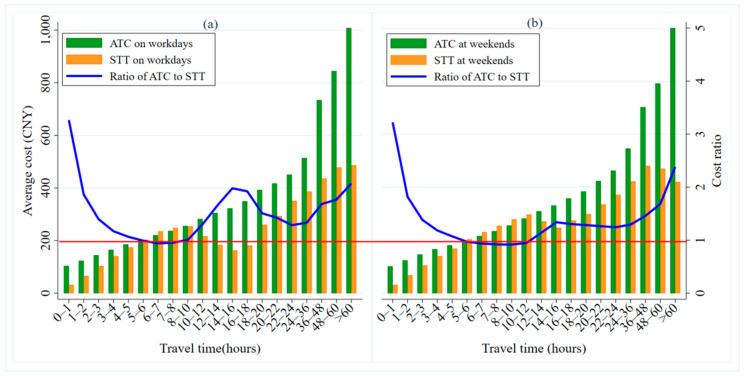
ATC and STT distributions over travel time on workdays and at weekends. (**a**) shows the ATC, STT and cost ratio of them over time on workdays, while the (**b**) is at weekends.

Figure 11 shows the distributions of ATC and STT over different travel durations, on workdays and weekends. Generally speaking, whether it is a workday or not, the longer the travel time is, the higher the ATC is, while the STT shows a trend of increasing at first, then decreasing, and finally, increasing again. The ATC is higher than the STT in most of the travel duration buckets, which indicates that taxi passengers have more cost advantages. When the travel time is shorter (less than 1 h), the cost advantage of taxis is relatively large, with an ATC about 3.2 times the STT. When the travel time is 1–24 h, the ATC is about 1.4 times the STT. When the travel time is longer than 24 h, the ATC is twice the STT.

Whether it is a workday or not, with an increase in travel time, the cost ratio of car-sharing to taxis first decreases and then, increases (as shown by the blue line in Figure 11a,b), peaking at 14–18 h. When the travel time is 5–12 h, the cost ratio drops below 1 (as shown by the red horizontal line in Figure 11a,b), indicating that car-sharing has become the cheaper mode of travel. However, there are significant differences between workdays and weekends when it comes to journeys of between 14 and 18 h. On workdays, the cost advantage of taxis over car-sharing is much larger. The ATC is about twice the STT. On the weekends, the cost advantage is smaller, with the ATC 1.3 times the STT. This is mainly because longer duration journeys are mostly multi-objective, and therefore, segmented trips that would require multiple taxis to complete. Moreover, taxis may be in short supply due to the large number of orders on workdays, making waiting times longer.

On workdays and weekends, we have found that car-sharing is a more economical way to travel for medium travel times, through a comparative analysis of ATC and STT. With long travel times, meanwhile, people are more likely to choose taxis. The reasons are as follows: on the one hand, medium and short distance journeys are dominated by multi-objective and segmented business activities (on workdays) or leisure and entertainment activities (on the weekend). These are mostly round-trip journeys. Thus, the more flexible mode of car-sharing, similar to private cars, becomes a better choice for travelers. On the other hand, long-duration journeys are generally long distance or have long stopping times. Taking one or more taxis to complete the journey does not require payment for the pick-up costs, return costs, and parking fees incurred by car-sharing travel.

### 4.4. Comparison of ATC and STT in the Peak and Off-Peak Traffic Periods

**Figure 12:**
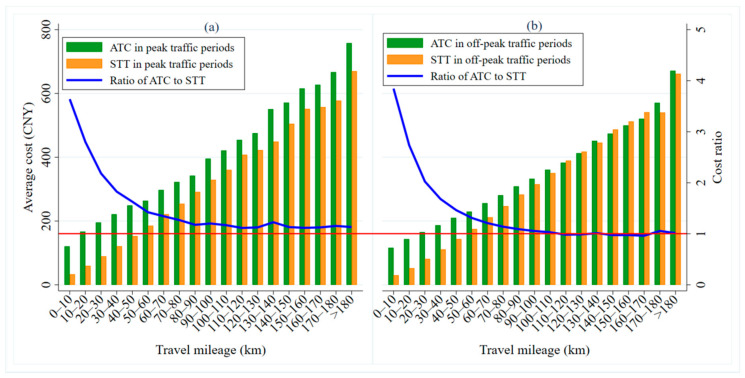
ATC and STT distributions over travel mileage during peak and off-peak traffic periods. (**a**) shows the ATC, STT and cost ratio of them over mileage during peak traffic periods, while the (**b**) is during off-peak traffic periods.

The travel costs of car-sharing and taxi are also affected by peak and off-peak traffic periods. Figure 12 shows the distribution of ATC and STT across different travel mileages in the peak and off-peak traffic periods. On the whole, in both the peak and off-peak traffic periods, the greater the mileage is, the larger the ATC and STT are. Moreover, with an increase in travel distance, the cost ratio of car-sharing to taxis gradually decreases (as shown by the blue broken line in Figure 12a,b). However, the cost ratio during off-peak traffic periods for long distance journeys is relatively small compared with that during the peak traffic periods.

In the peak traffic periods, the ATC is higher than the STT over different travel distances (as shown in Figure 12a), which indicates that travelers gain a cost advantage by taking taxis. When the travel distance goes beyond 100 km, the cost ratio gradually approaches 1 and then, stabilizes (as shown by the red horizontal line in Figure 12a). For example, when traveling short distances (less than 10 km), the cost advantage of a taxi is relatively large, with the ATC being about 3.6 times the STT. When traveling long distances (more than 100 km), taxis’ cost advantage falls, with the ATC being about 1.1 times the STT. During off-peak traffic periods (as shown in Figure 12b), when the travel distance is 110–170 km, travelers gain a cost advantage from car-sharing (the ATC is lower than the STT). However, for other travel distances, especially below 100 km, taxis are more cost-advantageous. When the travel mileage exceeds 100 km, the cost ratio drops to 1 and then, stabilizes (as shown by the red horizontal line in Figure 12b). To be specific, when traveling short distances (less than 10 km), travelers have the greatest cost-advantage from taking taxis, with the ATC about 3.9 times the STT. When traveling long distances (more than 100 km), the ATC and STT are basically equal. 

From the comparative analysis of the ATC and STT in peak and off-peak traffic periods, it can be seen that it is more economical for travelers to use taxis for various travel distances. However, for long journeys in peak traffic periods, taxi and car-sharing costs are about the same. This is mainly because travelers prioritize commuting or other travel activities with a single objective. Thus, on the one hand, car-sharing, because of the use of stations, may increase the commuters’ time cost. On the other hand, the traffic congestion at rush hours increases the leasing time and traveler’s total travel costs. Outside of rush hours, travelers are mainly engaged in multi-objective business activities, tours, and other travel activities. Although car-sharing is a more convenient and flexible way to travel, the long rental fees will be high and it may be hard to find space to park.

**Figure 13:**
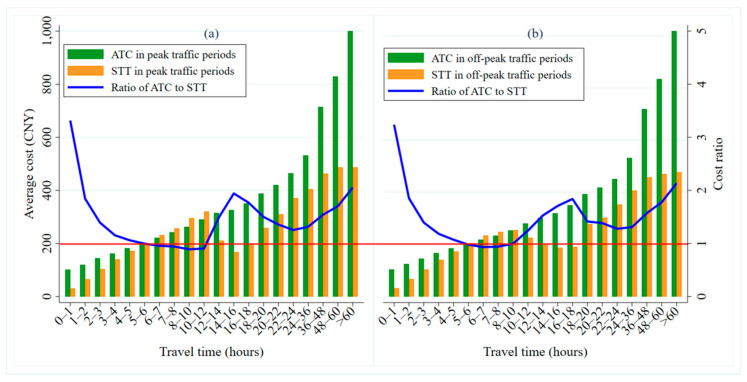
ATC and STT distributions over different travel times during the peak and off-peak traffic periods. (**a**) shows the ATC, STT and cost ratio of them over time during peak traffic periods, while the (**b**) is during off-peak traffic periods.

Figure 13 shows the distributions of ATC and STT over different travel time intervals in the peak and off-peak traffic periods. Overall, ATC is always higher than STT, indicating that travelers have cost advantages when using taxis to travel. The longer the travel time of the traveler, the higher the ATC is, while the STT shows a trend of first increasing, then decreasing, and finally, increasing again. Specifically, when traveling for a short duration (less than 1 h), the cost advantage of traveling by taxi is the largest, with the ATC about 3.3 times the STT. At the same time, with the growth in travel time, the cost ratio of car-sharing to taxis presents a trend of first decreasing and then, increasing (as shown by the blue line in Figure 13a,b). The peak occurs between 14 and 18 h, when the ATC is about twice the STT. For medium-duration journeys, the cost ratio is about 0.95 (as shown by the red horizontal line in Figure 13a,b). In this case, travelers have a cost advantage from using car-sharing. In conclusion, through the comparative analysis of ATC and STT, we have found that for medium-length travel times, car-sharing is more economical, while for short and long travel times, people see a cost benefit from taking taxis.

To sum up, car-sharing and taxi have different cost competitive advantages under different travel mileage and travel time characteristics. However, taxis have the advantage of travel costs on the whole. The key is the current market pricing strategy. Taxis are divided into different charging strategies according to the mileage, periods, etc. Car-sharing should also be priced separately according to their different travel mileage and rental time, so as to broaden the application scope of car-sharing and promote the further development of the industry. Note that our calculation results are related to the parameter values of the model. When different parameters are taken, the calculation results are different. But the overall difference is not large, and the robustness of the model is high. The calculation results of different parameter values are shown in Appendix A.

## 5. Discussion

Having compared and analyzed the travel cost advantages of using car-sharing and taxis in different travel scenarios, we offer the following discussion.

### 5.1. Car-Sharing

The emergence of car-sharing has provided a new mode of transportation for medium- and long-distance and urban fringe travel. However, according to our calculation and comparison results, car-sharing only has cost advantages for long-distance journeys, but has no cost advantage for long travel times. The main reasons for this are the hourly charges for car-sharing and urban parking problems. Therefore, enterprises should, on the one hand, design different pricing mechanisms suitable for different models, regions, and leasing methods. On the other hand, they could give out coupons, discounts, packages, and other incentive policies to increase the cost advantages of car-sharing. Regarding parking problems in cities, the government should improve the parking policies and relevant regulations, classifying specific parking spaces for car-sharing. In addition, enterprises can set up car-sharing parking areas or intelligent parking garages in urban central areas, and encourage users to park shared cars in certain urban areas to facilitate travelers’ use of them and enhance users’ experience and satisfaction. In some European countries where car-sharing is well developed, there are some policies about car-sharing parking. For example, at the beginning of the establishment of Bollore Group, the Government of Paris provided land transfer policies and capital loans, and implemented preferential parking policies. In some Italian cities, car-sharing is being promoted by offering free parking at fee-paying car parks in the city center [55].

Users are direct participants in car-sharing, and the development of car-sharing cannot be separated from users’ experience and trust. Travelers should reduce their car ownership, strengthen their right for use, and encourage other people of all ages to actively respond to policies related to urban low-carbon transportation and sustainable development. Governments need to enhance users’ awareness of environmental protection and change their consumption concepts. In addition, travelers’ choices of transportation mode (car-sharing or taxi) and travel cost structures are directly affected by pricing rules, the network layout, and how car-sharing operations are managed. Therefore, consumers, governments, and enterprises need to strengthen their cooperation in order to promote the further development of the car-sharing market. On the one hand, enterprises and governments should focus on consumers and provide them with quality services. For example, in the face of competition from public transport, taxis, and other travel modes, car-sharing enterprises can establish good cooperative relations with bus and taxi companies under the coordination of the government. They can draw on each other’s strengths to build a package of mobile service plans. They can set up pick-up and return stations near subways and bus stations to make it easier for travelers to transfer between modes. On the other hand, enterprises can provide convenience for travelers by improving the car rental network system and application software. For example, they can take full account of the layout of the charging stations to improve the mileage of new-energy vehicles. They can also use a reasonable design and configuration of pick-up and return stations (the locations of the stations and the number of vehicles that are located in each station), so that customers can pick up and return cars quickly and easily. For example, the German company BMW has added a lot of new technologies to car-sharing, such as on-board WiFi, intelligent voice, fingerprint intervention, alcohol test, face recognition, and other functions, which can create a safer, more convenient and comfortable driving environment for users. ChargeNow has optimized service and digital charging solutions to give customers easy access to the current world’s largest public charging pile network with 143,000 charging piles to easily recharge and pay for electric vehicles. By combining with exclusive parking lots in the city, it brings more convenience to users and improves their driving experience [56,57].

Another option is to provide ordinary, luxury, business, and other different types of vehicles to meet the needs of different levels of consumers, and expand the service market. In foreign countries, there are many precedents for car-sharing enterprises to expand the market. Zipcar has partnered with the State of New York to rent idle cars to government employees through Zipcar’s rental software, which has helped reduce costs and save energy and emissions. In addition to working with the government, Zipcar has pioneered a way to divide its other target customers into corporate users, individual users, and college students. By receiving online registration information from these users, it offers membership cards in the hope of cultivating long-term customers [58]. Finally, after using car-sharing services, users could be encouraged to give feedback to the enterprises and put forward suggestions for improved service quality.

### 5.2. Taxis

In the process of urban traffic development, the indispensable taxi can provide high-quality travel services for people. It has the advantages of being convenient, comfortable, available all day, etc. In recent years, against the background of “Internet+”, the emergence of car-hailing apps has greatly facilitated people’s travel. More and more taxi drivers have joined the online car-hailing trend and begun using apps to receive orders. At the same time, taxis can also provide the service of stopping when waved down at the roadside. These joint operations of taxis provide a very big development space and more efficient and faster travel services. However, car-sharing provides people with a more environmentally friendly and convenient way to travel. It is not only replacing private cars to a certain extent, but is also gradually influencing the taxi market and bringing new opportunities and challenges to its operational management and development. Many young people with driving licenses are choosing green car-sharing, which provides users with a comfortable and personalized travel experience. However, the convenience is also restricted by many factors, such as station distribution, parking locations, vehicle failure, and traffic accidents. On the other hand, the large-scale development of taxis brings many problems, such as low traffic efficiency in peak traffic periods and the frequent phenomenon of urban traffic congestion. However, taxi vehicles are uniformly managed and deployed by the taxi companies, and all drivers are trained and audited. In addition, with the standardization and improvement of the Internet car-hailing platform in recent years, the taxi market is gradually becoming more efficient and standardized.

From the perspective of travel costs, car-sharing and taxis show different characteristics under different travel situations, which are complementary and competitive. Travelers who use car-sharing must meet certain higher requirements in terms of personal abilities, such as driving skills and proficiency in using software. However, travelers who take taxis have higher requirements in terms of the environment, such as time, place, weather, etc. Taxis meet travelers’ travel needs over short time durations and short distances. Car-sharing brings the possibility of long-distance travel. For medium-distance and medium-duration travel, the two modes compete and complement one another more. Therefore, under the environment of the sharing economy, relevant government departments should improve the following three aspects. First, according to the urban position and needs, the taxi industry should reform by resorting to the Internet platform. Taxis, public transport, car-sharing, and other passenger transport services need to better develop market segments and implement differentiated management. Second, a customer evaluation system should be established to improve the service. Meanwhile, the service platform should take responsibility for management and establish a complaint-reporting mechanism. Third, given their contribution to an important mode of urban transportation, relevant government departments must take effective measures to avoid the continuous loss of taxi drivers. Through the reasonable establishment of a price mechanism and incentive measures, they need to increase the drivers’ incomes and protect their right.

From our results, we have found that taxis have greater cost advantages over short distances and short durations of journeys compared with car-sharing. This is because people favor taxis because of the convenience and speed of hailing them. However, taxi operations under such travel characteristics will lead to traffic congestion, vehicle shortages in the peak traffic periods, and unequal resource allocation.

To solve these problems and realize the mutual benefits and complementarity of taxis and car-sharing, on the one hand, relevant government departments should strengthen the operation and management of the taxi market and make innovations in its management. For example, according to the actual needs of the market, the government should strictly control the number of taxis to reduce the use of traffic resources and effectively alleviate pressure on urban traffic. In view of the large difference in taxi distribution density in peak traffic periods, enterprises and governments should employ professionals to analyze the supply and demand imbalance in the taxi market in depth. By reducing the amount of taxis scientifically and reasonably, they can maintain healthy competition in the whole industry. On the other hand, taxi companies should change the traditional business model and participate in the competition of the sharing economy. For example, they could try offering car-sharing services or offering car rental services through which users could find and rent taxis for a long time via the internet.

## 6. Conclusions

Understanding the competitive advantages of car-sharing and taxis from the perspective of travel costs is of great theoretical and practical significance for improving the management of urban traffic. Taking Beijing as an example, this paper uses GPS trajectory data from car-sharing to design a travel cost framework of car-sharing and taxis. The travel time costs and parking costs are quantified as the money cost, and the travel costs of the two modes are calculated through the construction of a model. By comparing the cost ratio for travelers of car-sharing versus taxis under different travel characteristics, this paper draws a series of valuable conclusions, as follows:

In terms of the users of car-sharing, they are mainly 25–34 years old and male. The quantity of orders of car-sharing decreases gradually with the increase in travel mileage and travel time. Most orders are for medium and short mileages and times. From the perspective of administrative and functional areas, the car-sharing orders are mainly distributed in Chaoyang, Haidian, Fengtai, and other main urban areas. In addition, they happen mainly in functional areas with commercial residences, science, education and culture, and companies. In view of this, enterprises should put vehicles in the developed urban areas to meet the different mileage and time demands of young males, and provide corresponding facilities (such as stations and parking lots).

The cost advantages of car-sharing and taxis show different characteristics on different days and at different times of day. The longer the travel distance and time, the higher the ATC and STT. Compared with car-sharing, taxis are more cost-advantageous for short distances and long durations, on workdays and weekends. Compared with on workdays, car-sharing is more cost-advantageous for long-distance journeys at weekends. When consumers travel for short or medium time periods, car-sharing is more economical every day. Compared with the off-peak traffic periods, it is more cost-advantageous for travelers to take taxis for different travel mileages and for both short and long durations in the peak traffic periods. Whether in the peak or off-peak traffic periods, car-sharing is more economical when traveling for a medium length of time. In addition, the cost ratio of car-sharing to taxis presents a similar trend in both peak and off-peak traffic periods. Therefore, we can speculate that these two travel modes are relatively little affected by the peak traffic periods. 

Exploring the competitive advantages in terms of the travel costs of car-sharing and taxis across time and space is of certain guiding significance. This can provide a reference for the formulation of relevant government policies and the adjustment of the operation and management of enterprises, and influence consumers’ choice of travel modes and travel concepts. However, this paper has the following limitations, which will be further studied and improved in the future: First, in measuring the travel costs of car-sharing, we only use the mileage charge plus the time charge. In practice, however, every car-sharing company has a variety of charging methods, including packages, monthly packages, coupons, etc. This will have a big impact on the use of car-sharing. Therefore, in our next work, we will study rental behaviors under different charging methods. Second, this paper focuses on data for car-sharing to compare the cost ratio of car-sharing to taxis. However, the car-sharing data have their own travel attributes, which will influence the analysis of users’ travel characteristics. In the future, we will use taxi data to calculate the actual travel costs of taxis and to simulate the travel costs of car-sharing, and compare the cost advantages with the results in this paper.

## Figures and Tables

**Table 1:** Sample data fields for a car-sharing order.

Data Field	Numerical Example
Order ID	17070107061720035940
Pick-up time stamp	1 July 2017 07:11:00
Return time stamp	1 July 2017 22:36:34
Pick-up station	Ground parking lot of Guantong Jianhui Hotel
Return station	China Cultural Building ground parking lot
CAR_ID	car_20003dmb2tzt

**Table 2:** Sample data fields for a car-sharing tracking record.

Data Field	Numerical Example
CAR_ID	car_20003dmb2tzt
DEVICE_NO	116232100003477
Time stamp	1 July 2017 07:52:56
Longitude	116.340704° E
Latitude	39.875464° N
Instantaneous speed	19.70 km/h

**Table 3:** Standard charges for Beijing parking.

λ	Region	Parking Fee		
		Day (7:00 a.m.–19:00 p.m.)		Night (19:00 p.m.–7:00 a.m.)
		Within the First Hour	After the First Hour	
1	Region of I	CNY 2.5 per 15 min	CNY 3.75 per 15 min	CNY 1 per 2 h
2	Region of II	CNY 1.5 per 15 min	CNY 2.25 per 15 min	
3	Region of III	CNY 0.5 per 15 min	CNY 0.75 per 15 min	

**Table 4:** Standard charges for Beijing taxis.

Category	Fare
Base rate (0–3 km)	CNY 13
Mileage fee	2.3 CNY/km
Low speed fee	Below 12 km/h: add 2 km’s rental per 5 min during the rush hour, add 1 km’s rental during other times (excl. empty cruise fee).
Empty cruise fee	Over 15 km carrying passengers one way, add 50% of the basic unit price.
Night-time charge	23:00 (incl. 23:00) to 5:00 next day (excl. 5:00) operation: add 20% of the basic unit price.

**Figure A1:**
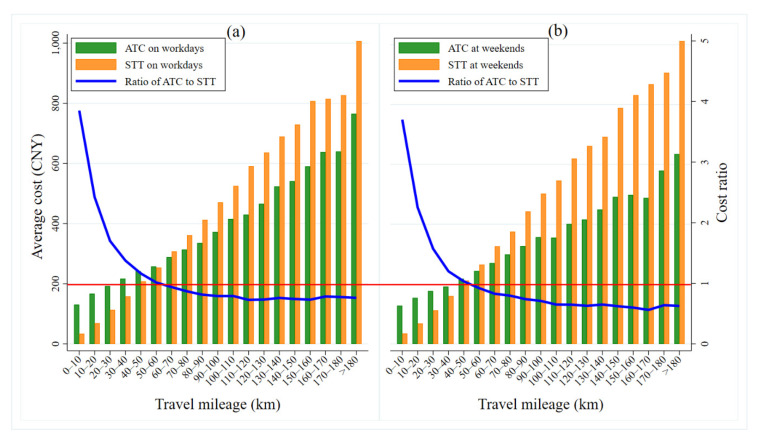
ATC and STT distributions by travel mileage on workdays and at weekends. (**a**) shows the ATC, STT and cost ratio of them over mileage on workdays, while the (**b**) is at weekends.

**Figure A2:**
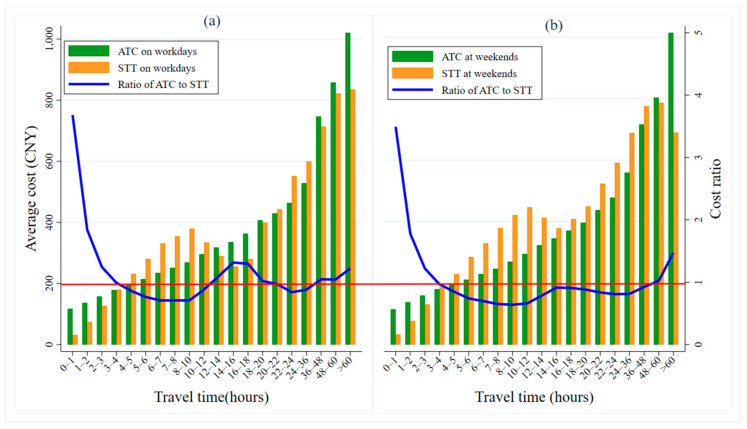
ATC and STT distributions by travel time on workdays and at weekends. (**a**) shows the ATC, STT and cost ratio of them over time on workdays, while the (**b**) is at weekends.

**Figure A3:**
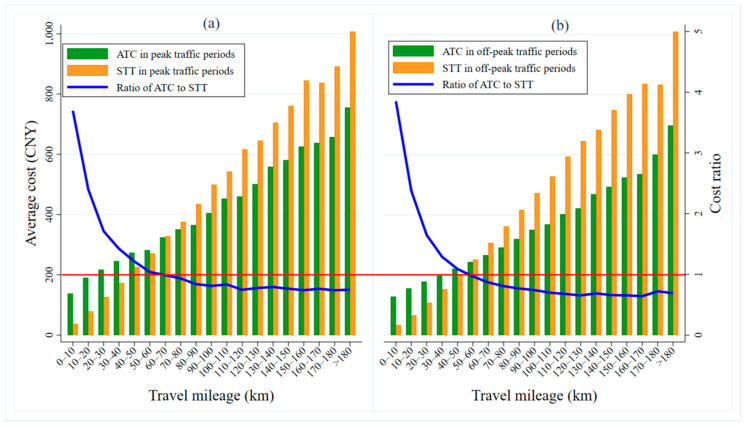
ATC and STT distributions over travel mileage during peak and off-peak traffic periods. (**a**) shows the ATC, STT and cost ratio of them over mileage during peak traffic periods, while the (**b**) is during off-peak traffic periods.

**Figure A4:**
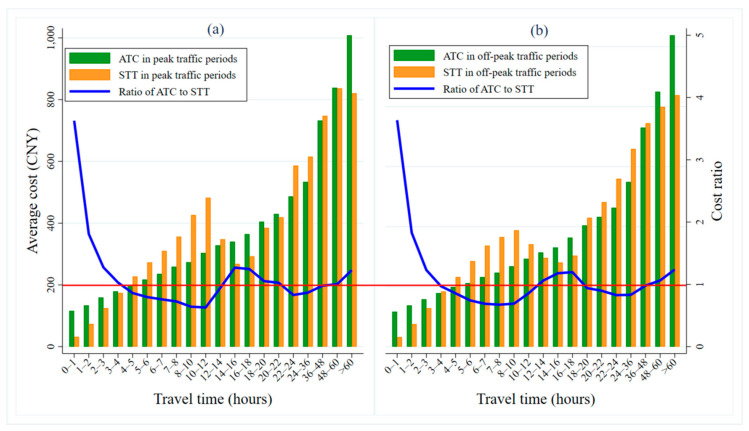
ATC and STT distributions over travel time during peak and off-peak traffic periods. (**a**) shows the ATC, STT and cost ratio of them over time during peak traffic periods, while the (**b**) is during off-peak traffic periods.

**Figure A5:**
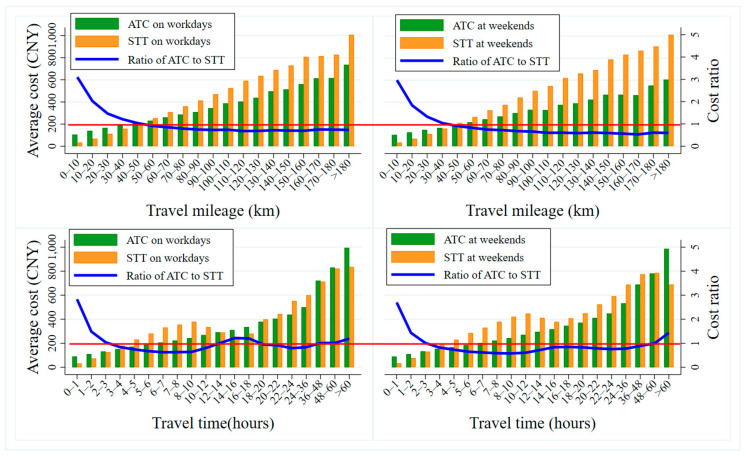
ATC and STT distributions over travel mileage and travel time during peak and off-peak traffic periods.

**Figure A6:**
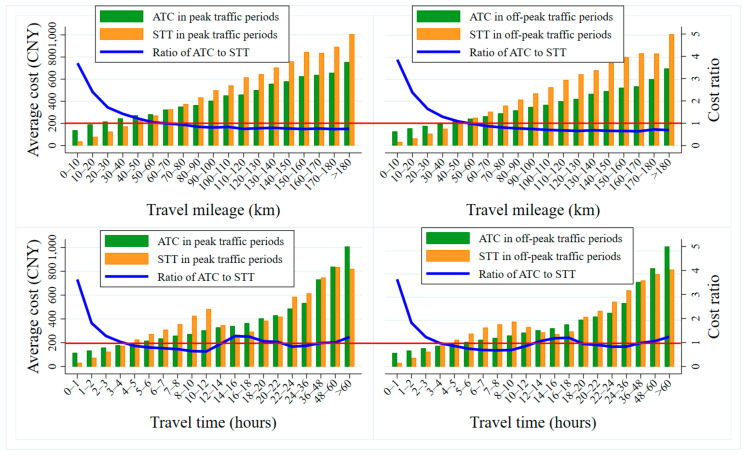
ATC and STT distributions over travel mileage and travel time during peak and off-peak traffic periods.

**Figure A7:**
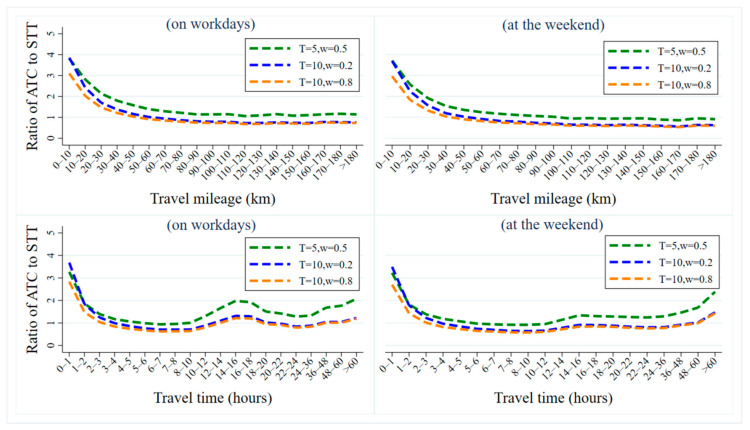
Ratio of ATC to STT of different parameter values on workdays and at weekends.

**Figure A8:**
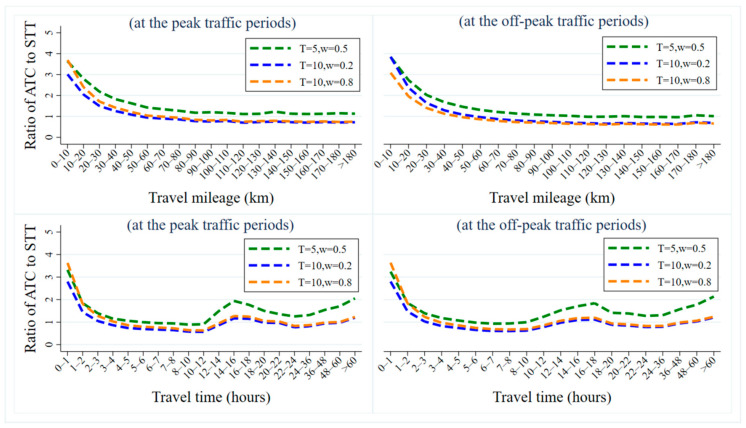
Ratio of ATC to STT of different parameter values during peak and off-peak traffic periods.

## Appendix A

Figure A1, Figure A2, Figure A3 and Figure A4 show ATC and STT distributions when *w_i_* = 0.2, *w_j_* = 0.8, *T* = 10 min. When the travel distance exceeds about 50 km, car-sharing starts to have a cost advantage, and the advantage at weekends is slightly larger than that on workdays (as shown in Figure A1). In terms of travel time (as shown in Figure A2), car-sharing has a wider range in cost advantages at weekends. If the rental time is 12–20 h on workdays, taxis have the cost advantage, while car-sharing has the cost advantage at weekends. When the travel distance exceeds about 60 km, car-sharing starts to have a travel costs advantage, which is slightly larger in the off-peak traffic period than in the peak period (as shown in Figure A3). In terms of travel time (as shown in Figure A4), car-sharing has a wider range in cost advantages during off-peak traffic periods. If the rental time is 12–20 h during peak traffic periods, taxis have the cost advantage, while car-sharing has the cost advantage during off-peak traffic periods.

Figure A5 and Figure A6 show ATC and STT distributions when *w_i_* = 0.8, *w_j_* = 0.2, *T* = 10 min. The analysis of the results was the same as Figure A1, Figure A2, Figure A3 and Figure A4.

Figure A7 and Figure A8 show the ratio of ATC to STT when *w_i_* = 0.5, *w_j_* = 0.5, *T* = 5 min and when *w_i_* = 0.2, *w_j_* = 0.8, *T* = 10 min respectively, where *w = w_i_* in the figures represents the proportion of riding to pick-up the car-sharing. No matter on a workday or not, the travel costs advantage trend of different parameter values is the same, and the values gap is not big. The above distribution features are also presented during the peak and off-peak traffic periods.

As we can see, when the parameters changed, so did the total costs of car-sharing and taxis. The cost-gap and cost-advantage of the two ways also change greatly. However, the cost distribution trend and cost-advantage trend of the two travel modes under different parameter values are the same, and the distribution between the travel peak traffic periods and off-peak periods is basically similar on workdays and at weekends.
